# Risk of Introduction of Bovine Tuberculosis (TB) Into TB-Free Herds in Southern Bahia, Brazil, Associated With Movement of Live Cattle

**DOI:** 10.3389/fvets.2018.00230

**Published:** 2018-10-02

**Authors:** Luciana N. Avila, Vitor S. P. Gonçalves, Andres M. Perez

**Affiliations:** ^1^Agência de Defesa Agropecuária da Bahia, Salvador, Brazil; ^2^Faculdade de Agronomia e Medicina Veterinária - FAV, Universidade de Brasilia, Brasilia, Brazil; ^3^College of Veterinary Medicine, University of Minnesota, St Paul, MN, United States

**Keywords:** epidemiology, risk assessment, bovine tuberculosis, animal movements, surveillance, Brazil

## Abstract

Bovine tuberculosis (TB) is a zoonotic disease, endemic in Brazil, with an impact on public health and trade. TB causes direct and indirect financial losses to infected farms and regions. Our study aimed at quantifying the risk of introduction of TB into TB-free herds of southern Bahia, Brazil, via movement of live cattle from other regions of the State. Results suggest that the annual risk of introducing TB into free farms of southern Bahia, either through legal or illegal trade of live cattle, is very low, varying, on average, between 0.001 and 0.006 depending on the region of origin of the animals. Noteworthy, illegal movements accounted for 90% of the risk demonstrating the importance of compliance with official regulations. These results are useful to inform both veterinary authorities and farmers in making decisions related to the regionalization of the control for TB in the country, with the ultimate goal of eliminating this major zoonotic disease from an important dairy region of Brazil.

## Introduction

Bovine tuberculosis (TB), caused by *M. bovis*, is a zoonotic disease of chronic evolution, characterized by granulomatous lesions in cattle and buffalo with an impact on public health and trade. Milk and beef from countries in which TB is endemic may suffer restrictions on exports into disease-free regions, impacting on the national economy through price reduction of the commodity, and market loss (1–3). With the objective of reducing TB impact on human and animal health, the Brazilian Ministry of Agriculture and Livestock (MAPA) established, in 2001, the National Program for Control and Eradication of Bovine Brucellosis and Tuberculosis (PNCEBT). Control measures regulated in the PNCEBT include the voluntary certification of TB-free farms by regular testing-and-culling of cattle and subsequent restriction on animal movements from infected farms (1). Certification of TB-free farms could bring direct benefits to the producer, such as surplus value on milk sold and less strict testing requirements before selling and transporting cattle. However, the number of certified properties in Brazil is negligible, with only 798 TB-free certified farms in 2016, out of a total of about two and a half million cattle farms. The number of TB-free certified farms in Brazil is low because there are not enough direct financial benefits for the farmer and certification costs are high (4, 5). Compensation for culled animals is important in the initial phase of herd sanitation and the premium payment on milk would help farmers to achieve a return on the investment over time, none of which is so far available to the vast majority of cattle farmers in Brazil (5). Similarly, the adoption of a TB-free seal in milk packaging has been suggested as a way to add value to dairy products (6).

The State of Bahia had 22 TB-free certified farms when this study was conducted. Those were small-scale family farms, and, since 2015, these properties managed to install a dairy plant that works only with milk from TB-free properties. The dairy plant has a productive capacity of 10,000 L/day and produces pasteurized milk, yogurt, butter, and cheese. The project had the participation of the government of Bahia for the investment in the dairy plant and of the city council of the city of Uibaí for the purchase of the milk with a premium price, provided the milk is used to supply the needs of public schools. Such initiative boosted the region's dairy value-chain and provided an incentive to farmers, while promoting public health (7).

**Figure 1:**
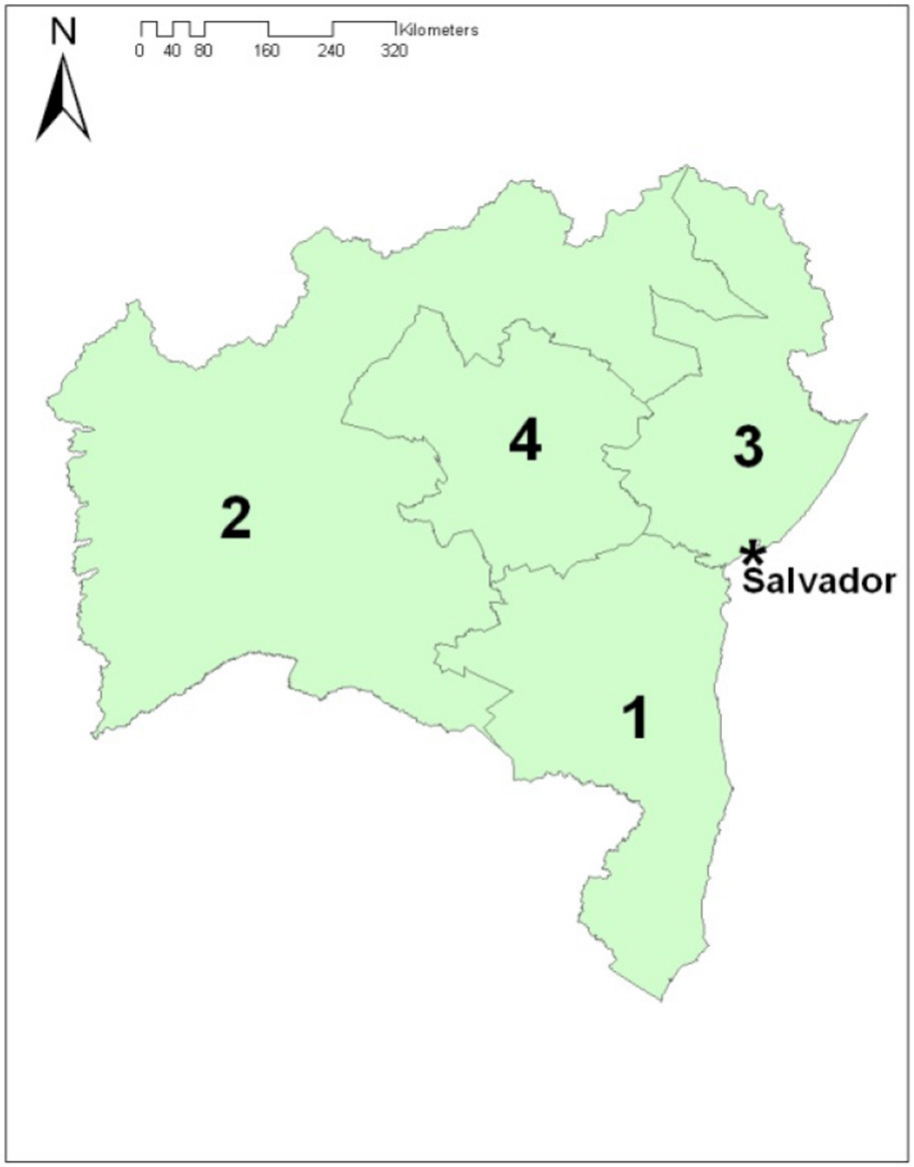
Map of the State of Bahia with the division in regions, referred to as south (1), west (2), north-east (3), and center (4) according to the production and animal trading practices. The location of the State's capital city, Salvador, is also depicted.

Data on the epidemiological dynamics of the disease in Bahia is still scarce, especially incidence data. A prevalence study conducted between October 2008 and November 2010 found a low farm-level prevalence (1.6, 95% CI: 1.0–2.6%) state-wide (8). Prevalence, although still low, was relatively higher (2%) in the southern region of Bahia (region 1, R1) (Figure 1), compared to other state regions. Southern Bahia is important for the State's economy because of the relatively higher concentration of intensive dairy farms. The same survey found that dairy and dual-purpose farms had higher risk of being TB positive than beef herds (OR: 9.72 and 6.66, respectively), and larger herds (more than 18 cows—aged ≥24 months) had higher risk (OR: 8.44) than the small ones. Those two factors were, possibly, indirectly related to differential rates of animal movements in to the herd (9).

The objective of our study was to quantify the annual probability of introduction of TB into free farms of southern Bahia (R1) through the movement of live animals from outside the region. Results will inform future policy decisions regarding the surveillance measures needed to protect free farms, or compartments of free TB farms, as the control and eradication program develops in southern Bahia.

This study is the first risk assessment applied to the control of animal diseases by the veterinary services of the State of Bahia and aims to support decisions on TB eradication. Risk assessment is a major component of risk analysis, as recommended by the World Animal Health Organization (OIE) and must be used to assist decision-making and guide public policy to support animal disease control (10). Ultimately, the methodology presented here may help to promote the routine application of risk assessments by the veterinary services of Brazil and elsewhere in the region.

## Methods

We carried out a stochastic quantitative risk assessment to estimate the risk for introduction of TB into free farms of southern Bahia (R1). Event trees, also referred to as scenario trees, were built for two scenarios, describing the risk associated with legal and illegal movement of live cattle from outside the region, respectively. We assumed that cattle are tested prior to legal movements, whereas illegally moved animals are not tested.

For the scenario of legal movements, risk was quantified considering three independent probabilities, namely, the probability that the farm of origin was TB-infected (P1), the probability that there was an infected animal in the farm of origin (P2), and the probability that the diagnostic test does not detect the infected animal, which was modeled as 1 minus the sensitivity (Se) of the diagnostic test (P3), so that P3 = 1–Se.

The probability of introduction of at least one infected animal into a free-herd through legal movement of cattle (P_L_) was estimated as1–(1–P1 × P2 × P3)^nL^, whereas the probability of introduction through illegal movement of cattle (P_I_) was computed as 1–(1–P1 × P2)^nI^, assuming that animals would not be tested when illegally moved, and where nL and nI are the number of animals legally and illegally introduced into a modal free-herd of southern Bahia, respectively.

Probabilities (P1–3) were parameterized using data obtained from the literature and *ad hoc* Pert distributions. P1 was modeled considering the region-specific prevalence values estimated from a study conducted in Bahia between 2008 and 2010 (8). For each region of origin, the most likely, minimum, and maximum values were assumed to match the point estimate and 95% confidence interval of the prevalence estimated in that study, corresponding to 2.9 (1.5–5.5%), 0.3 (0.04–2.1%), and 0.6 (0.2–2.5%) for regions of origin R2, R3, and R4 (Figure 1), respectively. No movement of animals into southern Bahia from other Brazilian States was recorded in 2013.

P2 was modeled using survey data on within-herd prevalence from the States of Bahia (8), Minas Gerais (11), and Parana (12), supplied by the authors of such studies. Minas Gerais and Paraná were selected, in addition to Bahia, because farm size and management of dairy farms is comparable to those found in Bahia, and, together, there were 127 positive farms in surveys of the three states, which helps to estimate the uncertainty and variability associated with the value of the parameter. Consequently, minimum, most likely, and maximum values for P2 were assumed to be 0.025, 0.050, and 0.667, respectively. For P3, it was assumed that animals would be tested using the Comparative Cervical Tuberculin Test (CCT), which is the test recommended by the PNCEBT. The sensitivity of the skin test depends for a large part on the quality of bovine tuberculin, the training and supervision of the operator. These are highly variable. Minimum, most likely, and maximum values for the Se of the CCT were estimated as 0.5, 0.807, and 0.98, respectively (5).

The value of nL was obtained from the database of the official veterinary service of Bahia (ADAB), ranging between 0 and 2 per farm per year. Subsequently, nL was modeled using probabilities of 0.7, 0.2, and 0.1 for the values of nL = 0, 1, and 2, respectively. The value of nI was estimated by administering questionnaires to farmers in 136 municipalities of southern Bahia. Questionnaires were administered in groups of *n* = 12 questionnaires until finding that the median and mean value of the output remained relatively stable. Such stability was reached after administering 36 questionnaires, and a discrete distribution was assumed for nI with probabilities of 0.6, 0.1, 0.06, 0.05, and 0.01 for the values of 0, 1 and 2, 3, 4 and 5, and 6-to-9 cattle introduced per farm per year, respectively.

The final annual probability (PF) was estimated as PF = P_L_ + P_I_ – (P_L_ × P_I_). The model was run with 1,000 iterations, using the Monte Carlo method for simulations, implemented in the software @RISK v. 5.7.1 (Palisade Corporation, Newfield, NY, USA).

## Results and discussion

Results suggest a very low probability of introduction of TB into free-herds of southern Bahia via movement of live cattle (Table 1). The risk associated with illegal movement of animals was 15 times higher than for legal movements. It is important to note that the probability of introduction through legal movement of cattle was estimated as assuming pre-movement tested while the probability of introduction through illegal movement of cattle was calculated assuming that animals would not be tested when illegally moved. The model used data that were not stratified by production purpose (dairy, beef, or dual-purpose) because such stratification was not available to us.

**Table 1:** Annual probability (top 95 percentile of the probability distribution, and mean values in brackets) of introduction of TB-infected cattle into southern Bahia, stratified by region of origin (R2–R4) and route of introduction of animals (legal, PL, and illegal, PI).

	**P_L_**	**P_I_**	**Total**
R2	0.0004 (≤ 0.00119)	0.00569 (≤0.01701)	0.00609 (≤ 0.0182)
R3	0.00006 (≤ 0.00002)	0.00107 (≤ 0.00318)	0.00113 (≤ 0.0032)
R4	0.00011 (≤ 0.00034)	0.00160 (≤ 0.02580)	0.00171 (≤ 0.02614)
Total	0.00057 (≤ 0.00155)	0.00836 (≤ 0.04599)	0.00893 (≤ 0.04754)

We assessed the risk of TB introduction of infected animals into free-herds of southern Bahia because producers are interested in strengthening the disease control program in the region, with the ultimate objective of achieving TB eradication. Farms in the region are predominantly dairies, holding the largest herd of adult females (age ≥24 months) of the State, with ~2.8 million head. The region is characterized by extensive production (92.4%) and crossbreds (53.2%) and animals are mainly purchased directly from other properties in the region (85.1%) (8). Those features, which result in a relatively low contact with farms outside the regions, may explain, at least in part, the low prevalence of TB (2%), and the low risk for TB introduction into the region through animal movements. The results support the vision of regionalizing for the control of the disease in Brazil, to protect the status of southern Bahia, and, eventually, progress to the eradication of the disease in the region, given the low disease prevalence and low risk for introduction estimated here.

TB is a chronic disease, with relatively low transmission rate, especially in extensive livestock production systems. Those features may account for the low risk of introduction found and with persistence of the disease at the relatively low levels of prevalence observed in the region. However, disease eradication would require enhanced prevention of introduction of infected animals into the region, for which, the rate of illegal movements poses a major challenge. Increasing producers' awareness and compliance with existing regulations is of paramount importance for advancing the current control program into a scenario compatible with disease eradication.

A surveillance system to eradicate the disease in the state should consider a component of increased surveillance in dairy farms where the disease appears to have higher prevalence (8, 11, 12) and measures to prevent illegal movements. Additionally, surveillance activities based on a test-and-slaughter scheme need to be supported by compensation funds for test-positive animals.

A risk-based surveillance system for dairy herds may be implemented through targeted sampling for these properties in order to detect and clean the residual focus of the disease.

It is also important to adopt measures directed at controlling the disease in infected herds, where movements should be restricted. In addition, the need for risk-based surveillance should consider herds with a previous history of the disease, which is pointed out in several studies as an important risk factor for the occurrence of TB (13, 14). Our results indicate that the risk of introduction of infected animal into free herds of Southern Bahia is very low, despite the wide range of values used in the model to represent the uncertainty about sensitivity of the CCT (5). However, it should be stressed that the implementation of a test-and-slaughter policy in the region may warrant the use of the single intradermal test, which is more sensitive and may be the best method of choice for use in the final stages of TB control. The use of the comparative test results in a higher specificity, but will result in a lower sensitivity and, consequently, higher estimate of the risk, as calculated here. However, the risk estimated here was nil, supporting the use of this test.

Our study is the first risk assessment conducted in Bahia to evaluate the risk of animal disease spread. Routine adoption of risk assessments by the official veterinary services would help to generate information that allows for a better understanding of the dynamics of the disease and its epidemiological and productive characteristics. Ultimately, risk analysis may help to evaluate the impact of alternative strategies in preventing and controlling disease spread in a region. In developing countries and regions, including the State of Bahia and Brazil, simulation exercises, including risk assessments, are critical to optimize the use of scarce financial and human resources.

As in many other risk assessments, an important limitation of the study relates to the absence of estimates on the true number of legal and illegal movements. That limitation is either due to the absence of reliable data on animal movements, prevalence, diagnostic test accuracy, or the role of other factors, such as wild reservoirs, which could result in biased results. As with any other risk assessments, the model should be expanded or updated, as new information becomes available for the parameterization of the model.

## Conclusion

In conclusion, our results indicate that the risk of introducing TB associated with animal movements into Southern Bahia is very low. However, the prevailing epidemiological and socio-economic conditions are not compatible with short-term disease eradication, mostly, due to the risk posed by illegal movement of animals and the scarce financial incentives for farmers to adhere to herd sanitation and to conform to the legislation. However, based on the low prevalence of TB in the area and the limited risk of introduction from animal movements, a regionalization strategy could be considered if the appropriate policy was in place, with the ultimate objective of, eventually, eradicating the disease from the region.

## Author contributions

LA student and lead author of the article. AP and VG doctoral thesis directors and collaborators in data analysis and article writing.

### Conflict of interest statement

The authors declare that the research was conducted in the absence of any commercial or financial relationships that could be construed as a potential conflict of interest. The reviewer AB declared a past co-authorship with the author AP and states that the process nevertheless met the standards of a fair and objective review.
