# Corrigendum: Risk of Introduction of Bovine Tuberculosis (TB) Into TB-Free Herds in Southern Bahia, Brazil, Associated With Movement of Live Cattle

**DOI:** 10.3389/fvets.2021.789706

**Published:** 2021-11-11

**Authors:** Luciana N. Avila, Vitor S. P. Gonçalves, Andres M. Perez

**Affiliations:** ^1^Agência de Defesa Agropecuária da Bahia, Salvador, Brazil; ^2^Faculdade de Agronomia e Medicina Veterinária - FAV, Universidade de Brazilia, Brazilia, Brazil; ^3^College of Veterinary Medicine, University of Minnesota, St Paul, MN, United States

**Keywords:** epidemiology, risk assessment, bovine tuberculosis, animal movements, surveillance, Brazil

In the original article, the **Conflict of Interest** statement was incomplete. The corrected statement appears below.

“The reviewer AB declared a past co-authorship with the author AP and states that the process nevertheless met the standards of a fair and objective review.”

The authors apologize for this error and state that this does not change the scientific conclusions of the article in any way. The original article has been updated.

## Publisher's Note

All claims expressed in this article are solely those of the authors and do not necessarily represent those of their affiliated organizations, or those of the publisher, the editors and the reviewers. Any product that may be evaluated in this article, or claim that may be made by its manufacturer, is not guaranteed or endorsed by the publisher.

